# Fernblock® Upregulates NRF2 Antioxidant Pathway and Protects Keratinocytes from PM_2.5_-Induced Xenotoxic Stress

**DOI:** 10.1155/2020/2908108

**Published:** 2020-04-14

**Authors:** Pablo Delgado-Wicke, Azahara Rodríguez-Luna, Yoshifumi Ikeyama, Yoichi Honma, Toshiaki Kume, María Gutierrez, Silvia Lorrio, Ángeles Juarranz, Salvador González

**Affiliations:** ^1^Department of Biology, Faculty of Sciences, Autónoma University of Madrid (UAM) 28049, Madrid, Spain; ^2^Medical Affairs Department, Cantabria Labs, 28043 Madrid, Spain; ^3^Rohto Basic Research Development Division, Tokyo, Japan; ^4^Department of Applied Pharmacology, Graduate School of Medicine and Pharmaceutical Sciences, University of Toyama, Sugitani, Toyama, Japan; ^5^Instituto Ramón y Cajal de Investigación Sanitaria (IRYCIS), Madrid, Spain; ^6^Department of Medicine and Medical Specialties, Alcalá de Henares University, 28805 Madrid, Spain

## Abstract

Humans in modern industrial and postindustrial societies face sustained challenges from environmental pollutants, which can trigger tissue damage from xenotoxic stress through different mechanisms. Thus, the identification and characterization of compounds capable of conferring antioxidant effects and protection against these xenotoxins are warranted. Here, we report that the natural extract of *Polypodium leucotomos* named Fernblock®, known to reduce aging and oxidative stress induced by solar radiations, upregulates the NRF2 transcription factor and its downstream antioxidant targets, and this correlates with its ability to reduce inflammation, melanogenesis, and general cell damage in cultured keratinocytes upon exposure to an experimental model of fine pollutant particles (PM_2.5_). Our results provide evidence for a specific molecular mechanism underpinning the protective activity of Fernblock® against environmental pollutants and potentially other sources of oxidative stress and damage-induced aging.

## 1. Introduction

Air pollution is a growing challenge to public health worldwide and constitutes an emerging focus of research and surveillance for the World Health Organization [1]. Because of the role of the skin as a primary barrier against external sources of tissue damage, continuous exposure to these pollutants has a substantial negative impact on this organ and is precursory of premature skin aging, pigmentation, acne disorders, and psoriasis exacerbation, among others [2]. Specifically, PM_2.5_ provokes increased ROS and loss of organelle homeostasis in keratinocytes [3], has been associated with aggravated allergic dermatitis and eczema in children [4], and is precursory to inflammation, aging, androgenic alopecia, and skin cancer [5]. Thus, air pollution, solar radiation, and tobacco smoke constitute extrinsic skin-aging factors, leading to ROS production and the subsequent activation of oxidative stress responses. Skin antioxidant defense responses are effective against these exogenous sources of damage; however, chronic exposure, aging, or several concomitant pathologies can lead to decreased activation and increased oxidative damage, accelerating skin aging and skin cancer [6]. Prevention strategies including sun protection, skin barrier improvement, aryl hydrocarbon receptor (AhR) modulation [7], and increased skin tissue resistance through potentiation of natural detoxification pathways are target opportunities for skin protection [8]. Fully understanding mechanisms by which tissues confront these sources of xenotoxic stress and potential pharmacological opportunities to leverage on them are warranted.

Nuclear factor erythroid 2-related factor 2 (NRF2; also known as nuclear factor erythroid-derived 2-like 2, NFE2L2) is a basic leucine zipper transcription factor highly conserved in metazoans [9]. In nonstressed cells, the NRF2 protein is bound in the cytoplasm, ubiquitinated and rapidly degraded to low levels by the Kelch-like ECH-associated protein 1- (KEAP1-) Cullin 3 ubiquitin ligase complex. Generic insults provoking oxidative or electrophilic stress in cells inactivate the KEAP1/CUL3 complex, promoting nuclear translocation of accumulating NRF2, which in turn orchestrates the expression of different antioxidant enzymes (including most components of the glutathione de novo synthesis pathway and glutathione transferases and peroxidases) and detoxifying effectors (NAD(P)H Quinone Dehydrogenase 1 (NQO1), heme oxygenase 1 (HO-1), or Multidrug Resistant Proteins (MRPs)) in most cell types [10]. NRF2 constitutes an emerging, appealing target for therapeutic modulation in multiple pathologies [11]. Of note, NRF2 activity has been specifically associated with response to various environmental pollutants that potentially act as xenotoxins, including air PM_2.5_ [12, 13].

Fernblock® is a natural standardized aqueous extract from the leaves of *Polypodium leucotomos* [14]. The use of decoctions of this fern was widespread in traditional medicine amongst local indigenous populations in Central America against numerous ailments, and modern medicine has confirmed its notable potential as an active conferring skin-specific antioxidant activity and protection against sun radiation damage (including aging, hyperpigmentation, and DNA damage) [15]. However, while evidence supporting a boosting of endogenous antioxidant and xenobiotic stress systems in cells is highly relevant for the therapeutic potential of Fernblock® [16–18], our understanding of the molecular mechanisms by which this occurs is limited.

Here, we contribute evidence suggesting that Fernblock® is capable of upregulating the NRF2 pathway as assessed by different direct and indirect readouts in cultured human cells and that this dose-dependent activation correlates with its protective effect not only against UVB radiation but also against exposure to PM_2.5_. These observations suggest a potential for Fernblock® not only as a natural activity against the detrimental effects of a broad range of environmental sources of xenobiotic stress and aging but also as a potential tool for activating the NRF2 pathway.

## 2. Materials and Methods

### 2.1. Cell Culture and Treatments

The nontumorigenic human keratinocyte cell line HaCaT was used for *in vitro* studies (Cell Line Service, Eppelheim, Germany). Cells were subcultured in different plate formats according to assay (see below), in Dulbecco's modified Eagle's medium (DMEM) supplemented with 10% (*v*/*v*) fetal bovine serum (FBS), 50 units/ml penicillin, and 50 *μ*g/ml streptomycin, in an incubator at 5% CO_2_, 37°C, and 95% humidity. Rat adrenal pheochromocytoma PC12 cells were maintained in Dulbecco's modified Eagle's medium supplemented with 5% fetal calf serum and 10% horse serum. All cell culture reagents were purchased from Gibco Inc. (Paisley, UK). Human Epidermal Keratinocytes (NHEK; KURABO Co., Japan; Cat. No. KM4109, Lot No. 04644) were grown in HuMedia KG2 (KURABO Cat. No. KK-2150S) and assayed in HuMedia KB2 (KURABO Cat. No. KK-2350S). Human vaginal malignant melanoma cells (HMVII; European Collection of Authenticated Cell Cultures (ECACC), UK; Cat. No. 92042701, Lot No. 14B033) were grown and assayed in RPMI1640, supplemented with 10% FBS and penicillin-streptomycin. All cell culture reagents were purchased from Gibco Inc. (Paisley, UK).

Treatments as described in different experiments were applied on cultures at 50-60% confluence from 10 mg/ml stocks in all cases, and FBS supplementation was reduced to 1%. Fernblock® was provided by Cantabria Labs (Spain). A Standard Reference Material (SRM) for experimental modelling of air pollutants at PM_2.5_ was purchased from Sigma-Aldrich (UK; Diesel Particulate Matter 1650b) and was routinely sonicated to avoid aggregation for 30 min′ for immediate use during the next hour. Sulforaphane was purchased from Sigma-Aldrich (Japan; Sulforaphane S4441-5MG).

### 2.2. Reagents and Antibodies

Cell viability was measured using an assay based on MTT (3-(4,5-dimethylthiazol-2-yl)-2,5-diphenyltetrazolium bromide) (Sigma-Aldrich, St Louis, USA). The following primary antibodies were used in the performed immunofluorescence and western blot assays: mouse anti *α*-tubulin (Sigma-Aldrich, St Louis, USA), anti-beclin1 (Cell Signaling, #3738), anti-HO-1 (Cell Signaling, #70081), anti-LC3 (Abcam, Ab58610), anti-NQO1 (Cell Signaling, #3187), and anti-NRF2 (Abcam, ab89443), The secondary antibodies employed were mouse IgG-Alexa 488 and rabbit IgG-Alexa 546 (Invitrogen, Oregon, USA) and mouse IgG peroxidase and rabbit IgG peroxidase (Thermo Scientific, Rockford, USA). The markers used for the *in vivo* staining were monodansylcadaverine (MDC) for phagolysosomes and LysoTracker Green (LTG) for lysosomes. Hoechst 33258 (Sigma-Aldrich) was used for cellular staining.

### 2.3. Luciferase Assays for NRF2 Activity in PC12 Cells

Complementary oligonucleotides spanning a functional antioxidant response element (ARE) from the human NQO1 gene reference promoter sequence (-473 to -440) were annealed and ligated into the pGL4.27 vector (Promega, Madison, WI, USA). The resulting reporter vector was stably transfected onto PC12 using Lipofectamine 2000 (Invitrogen, Carlsbad, CA, USA) according to the manufacturer's instructions. Firefly activity in cell lysates from each assayed condition was measured in a luminometer using a Picagene LT2.0 Luminescence Reagent (Toyo Ink, Tokyo, Japan) according to the manufacturer's instructions. Results shown are derived as mean values and standard deviations from three independent experiments.

### 2.4. Assays for Studying UVB-Induced Damage in Human Keratinocytes

NHEKs were seeded onto 6-well culture plates at 300,000 cells/well. After 24 h spreading, cells were switched to KB2 medium supplemented with either vehicle (DMSO) or 31.3 *μ*g/ml Fernblock® for 24 additional hours. Cultures were then rinsed in PBS, irradiated with UVB (90 mJ/cm^2^) using a F1215 unit (Muranaka Medical Instruments Co. Ltd., Japan), and further cultured in KB2 for 6 h (cytokine expression assays), 24 h (cell counting and NRF2 activity assays), or 72 h (melanization assays).

Cell counting was automated from Hoechst 33342-stained samples using the ImageXpress Micro platform (Molecular Devices LLC, San José, CA, USA). For gene expression assays, total cell RNA was isolated using the RNeasy mini kits (QIAGEN) and reverse-transcribed using ReverTra Ace technology (Toyobo Co., Osaka, Japan). cDNAs were then quantitated by real-time PCR ^(-*ΔΔ*Ct^ method) using the ABI TaqMan™ Fast Advanced Master Mix (Thermo Scientific, USA) and the following ABI TaqMan™ probes for human sequences: catalase (CAT) (Hs00156308_m1), glutathione peroxidase (GPX) (ABI TaqMan Probe: Hs00829989_gH), GPX 4 (ABI TaqMan Probe: Hs00157812_m1), HO-1 (ABI TaqMan Probe: Hs00157965_m1), NQO1 (ABI TaqMan Probe: Hs00168547_m1), interleukin (IL)6 (ABI TaqMan Probe: Hs00174131_m1), and IL8 (ABI TaqMan Probe: Hs00174103_m1).

Melanin production was assayed as follows: KB2 72 h-conditioned medium from NHEKs supplemented and irradiated as indicated (see above) was diluted 1 : 1 with HMVII medium and added to a monolayer of HMVII cells 24 h postseeding. After 48 h culture for uptake, HMVII cells were trypsinized, pelleted, and treated with 2 N NaOH for 40 min at 80°C. Melanin content was measured by absorbance within a 405 nm–620 nm range using a plate spectrophotometer, corrected for cell viability (as assessed by trypan blue exclusion staining), and normalized to values observed in cells exposed to supernatants from nonirradiated cells (“control”).

### 2.5. Cellular Toxicity in PM_2.5_-Exposed HaCaT Keratinocytes

Toxicity of different concentrations of Fernblock® and PM_2.5_was inferred by the MTT colorimetric assay in HaCaT cells 24 and 48 h posttreatment. Briefly, cells were exposed after indicated treatment times to 50 *μ*g/ml MTT (3-(4,5-dimethylthiazol-2-yl)-2,5-diphenyltetrazolium bromide) at 37°C for 3 hours in the dark. Precipitated formazan was solubilized in DMSO (PanReac, Barcelona, Spain), and absorption was measured at 542 nm in a spectrophotometer (Spectra Fluor, Tecan). Cellular toxicity was expressed as the percentage of formazan absorption from the different treatment conditions compared to nontreated cells. Results shown are derived as mean values and standard deviations from three independent experiments.

### 2.6. Immunofluorescence Microscopy and Image Analysis

Active formation of phagolysosomal vacuoles was monitored by incubation of cells grown on coverslips with MDC for 10 min. Lysosomal compartment was decorated with LysoTracker Green™ (Molecular Probes), following the manufacturer's instructions. After briefly washing cells with PBS, slides were immediately mounted for image acquisition under UV or green excitation light.

For immunostaining, cells grown on the coverslips were fixed in a 3.7% formaldehyde solution in PBS for 10 min, washed with PBS 1X three times, and permeabilized with Triton X-100 0.1% in PBS during 30 min in agitation. Samples were incubated with primary antibodies for 1 h at 37°C, inside a humid chamber. After washing with PBS, cells were incubated with secondary antibodies for 45 min at 37°C. Nuclei were counterstained with Hoechst 33258. Coverslips were then washed with PBS and mounted with ProLong® antifade mounting medium (Molecular Probes). Images were acquired on an Olympus BX61 epifluorescence microscope equipped with filter sets for fluorescence microscopy: ultraviolet (exciting filter BP360-390), blue (exciting filter BP460-490), and green (exciting filter BP510-550), coupled to an Olympus CCD DP70 digital camera. LC3 immunofluorescence was quantified using ImageJ from at least fifty cells from each condition. NRF2 activation was assessed by separately computing nuclear and cytoplasmic intensities, from fifty cells from each condition. Figures were prepared using the Adobe Photoshop CS5 extended version 12.0 software (Adobe Systems Inc., USA).

### 2.7. Western Blotting

Cells were lysed in RIPA buffer (150 mM NaCl, 1% Triton X-100, 1% deoxycholate, 0.1% SDS, 10 mM Tris-HCl pH 7.2, and 5 mM EDTA), supplemented with phosphatase inhibitors and protease inhibitor cocktail tablets (Sigma-Aldrich, St. Louis, MO). Protein concentration was measured by BCA assay (Thermo Scientific-Pierce, Rockford, USA). Protein samples were subjected to SDS-PAGE and blotted to Immobilon-P PVDF membranes (Millipore Co., Massachusetts, USA). Membranes were blocked in PBS-tween 0.1% with 5% nonfat dried milk for 1 h at 25°C and then incubated with primary antibodies overnight at 4°C. After extensive washing with PBS-tween 0.1%, membranes were incubated with peroxidase-conjugated secondary antibodies. Signal was developed by chemiluminescence (ECL, Amersham Pharmacia Biotech, Little Chalfont, UK) and acquired on a ChemiDocTR XRS+ high definition system (Bio-Rad). Bands corresponding to the different proteins were digitalized employing the Image Lab version 3.0.1 (Bio-Rad Laboratories). This assay was performed at least three times for each target.

### 2.8. Statistical Analysis

Data are expressed as the mean value of at least three experiments ± standard deviations (SD). The statistical analysis was made using the statistical package of the program GraphPad Prism 6. Statistical significance was determined using a *t*-test and analysis of variance (ANOVA), and *p* < 0.05 was considered statistically significant.

## 3. Results

### 3.1. Fernblock® Induces a NRF2-Dependent Transcriptional Pathway which Correlates with Its Protection against UVB Radiation

**Figure 1:**
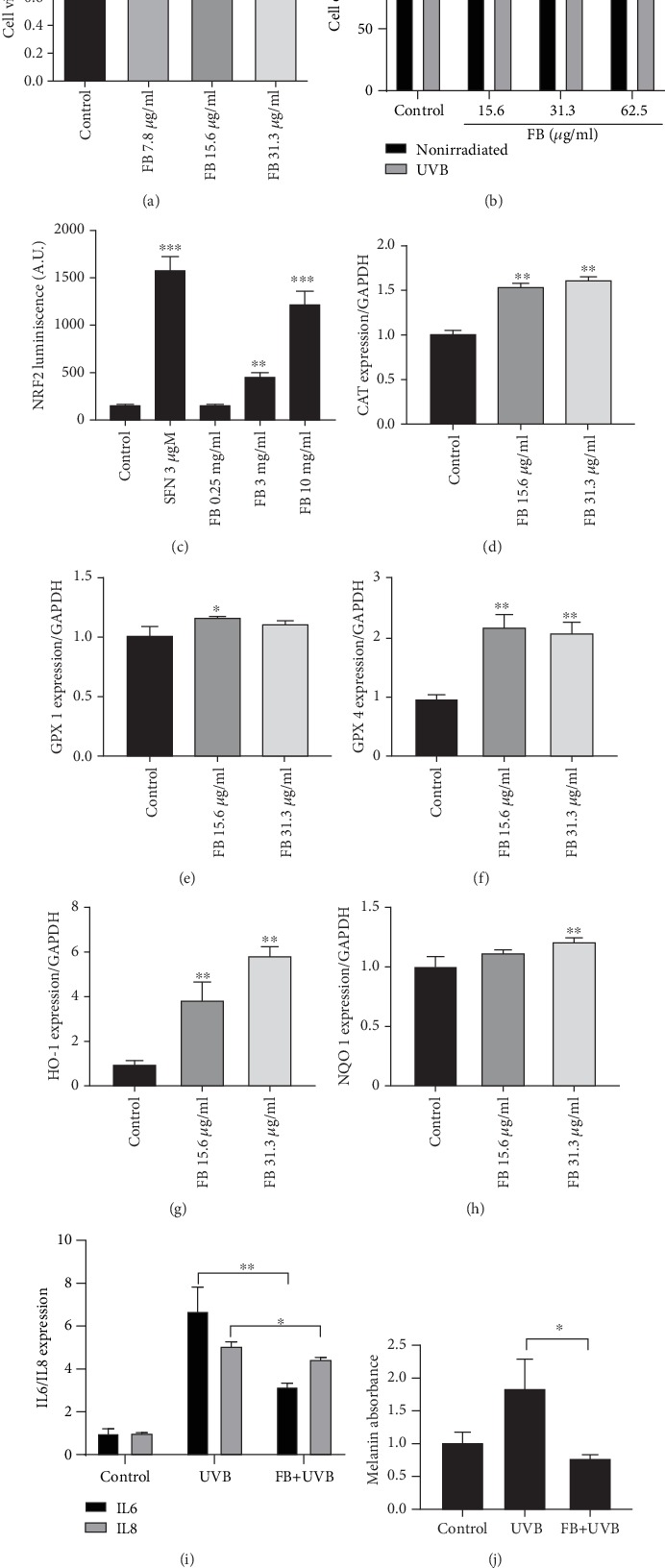
Fernblock® (FB) induces NRF2-dependent transcription activity and attenuates UVB-induced inflammation and melanization. (a) Fernblock® supplementation does not have a significant impact on cell viability as assessed by MTT assay. Data derived from 3 independent replicates and normalized to vehicle-treated samples (black bar, value: 1). (b) Fernblock® supplementation reverts the decrease in viability associated with UVB exposure in keratinocytes, as assessed by cell counts. The effect of UVB exposure (grey bars) is normalized to the corresponding nonirradiated samples (black bars, expressed as 100% viability) for each Fernblock® treatment group. Data are derived from 3 independent biological replicates. n.s.: nonstatistically significant; ^∗^ and ^#^*p* ≤ 0.05. (c) Fernblock® induces the activity of a synthetic NRF2-dependent luciferase minigene reporter. Stable PC12 cells bearing a NQO1-driven minimal promoter were treated as indicated (vehicle: DMSO; time: 24 h). Data are normalized to control, which is expressed as 100% signal. *n* = 3. (d–h) mRNA expression levels (^-*ΔΔ*Ct^ quantitation method) for indicated genes in human keratinocytes across indicated treatments were assessed by qRT-PCR using TaqMan technology. Data are normalized to the control (vehicle). *n* = 3. (i) mRNA expression levels for either IL6 (black bars) or IL8 (grey bars) across indicated treatment conditions in keratinocytes were assessed by qRT-PCR using TaqMan technology. *n* = 3. (j) Induction of keratinocyte melanization by the indicated treatments was assessed as detailed in Materials and Methods. Data are derived from absorbance across the visible spectrum and normalized to values from control samples. *n* = 3. ^∗^*p* ≤ 0.05, ^∗∗^*p* ≤ 0.01, ^∗∗∗^*p* ≤ 0.005, and ^∗∗∗∗^*p* ≤ 0.0001.

Previous studies demonstrated that Fernblock® is capable of exerting a protective effect on cells against UVB irradiation [15–19]. Thus, we first corroborated that Fernblock® treatment had no toxic effect against cell viability (Figure 1(a)), as well as its protective effect attenuating UVB-induced decrease in cell proliferation/viability, as assessed by normalized cell count (Figure 1(b)). However, the molecular mechanisms by which this occurs are not completely characterized. We decided to test the ability of Fernblock® to induce a major endogenous pathway deployed by cells to counteract oxidative and electrophilic stress: the NRF2 pathway.

First, we employed an established cell line of ectodermal lineage (PC12 pheochromocytoma cell line, widely used as a model for studying NRF2 activity [20]), stably expressing a luciferase reporter under the control of a promoter fragment derived from the canonical NRF2 target NAD(P)H Quinone Dehydrogenase 1 (NQO1). Several studies report that the exposure to the organosulfur compound sulforaphane leads to increased transcription of nuclear NRF2 and downstream cytoprotective genes [20–22]. Of note, Fernblock® induced the reporter in a concentration-dependent manner, suggesting that it is capable of upregulating this protective pathway in cells (Figure 1(c)).

We then investigated whether this effect also occurs in keratinocytes and whether it is associated with specific protection from exposure to UVB radiation. qRT-PCR assessment of mRNA levels for several bona fide targets of NRF2 (CAT, GPX 1 and 4, HO-1, and NQO1) revealed that Fernblock® induces the transcription of all these genes in human keratinocytes in a concentration-dependent manner (Figures 1(d)–1(h)). Similar antioxidant effects have been previously observed in a different *in vitro* model with a sulforaphane combination.

Importantly, in line with previous results, these treatment routines with Fernblock® protected keratinocytes from UVB radiation, decreased UVB-dependent induction of inflammatory cytokines IL6 and IL8 (Figure 1(i)), and significantly reduced the induction of melanin production (Figure 1(j)). Because NRF2 is a well-established prosurvival and anti-inflammatory pathway [10, 11], we consider the association of NRF2 induction with protection from UVB-induced damage in cells exposed to Fernblock® to be functionally relevant.

### 3.2. Fernblock® Protects Human Keratinocytes from Damage Induced by Fine Particle Pollutants (PM_2.5_) in an *In Vitro* Model

**Figure 2:**
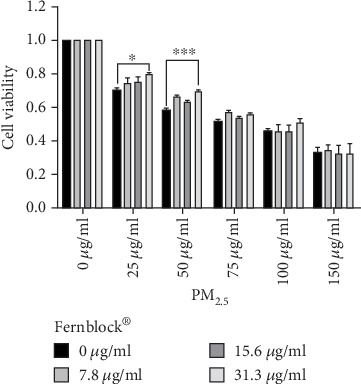
Fernblock® protects keratinocytes from PM_2.5_-induced toxicity. MTT assay was performed on HaCaT samples across indicated treatments on PM_2.5_, Fernblock®, or combinations of both compounds (see Materials and Methods). All data are expressed as normalized to untreated samples (leftmost bar group of graph). *n* = 3. ^∗^*p* ≤ 0.05 and ^∗∗∗^*p* ≤ 0.005.

The protective effect of Fernblock® against a broad spectrum of solar radiation is well established [16–18]. We considered whether this protective mechanism might also operate in a different condition leading to severe cell stress: high exposure to fine particle pollutants (PM_2.5_). Exposure of keratinocytes to a SRM of PM_2.5_ decreased cell viability in a concentration-dependent manner (Figure 2). On the other hand, simultaneous addition of Fernblock® in concentrations previously shown to induce NRF2 and protect from UVB-induced cell damage partially reverted toxicity associated with low to moderate concentrations of PM_2.5_ (25-50 *μ*g/ml; Figure 2), although higher PM_2.5_ doses were not counteracted in this *in vitro* model (Figure 2). The selected PM_2.5_ dose was 50 *μ*g/ml for all experiments. Fernblock® doses did not have any intrinsic effect on cell viability in the absence of PM_2.5_ (Figures 1(a) and 2).

### 3.3. Fernblock® Increases Basal and PM_2.5_-Induced Autophagy in Keratinocytes

**Figure 3:**
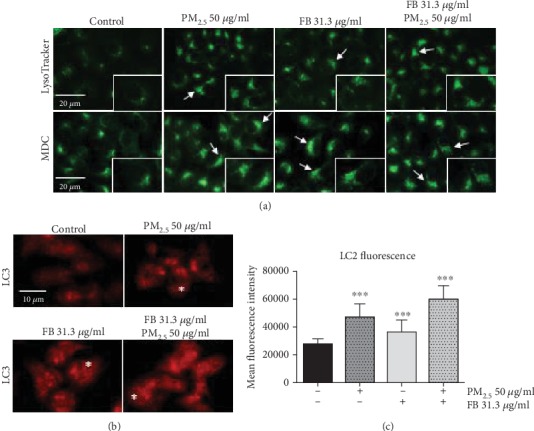
Fernblock® (FB) induces autophagolysosomal activity in keratinocytes *per se* and boosts autophagy flux in PM_2.5_-treated cells. (a) Expansion of the lysosomal compartment (LysoTracker Green™, top panel row) and density of autophagolysosomal vacuoles (MDC, lower panel row) were acquired by immunofluorescence microscopy across indicated treatments in HaCaT cells. Scale bar for whole-field images (20 microns) and 3x enlarged insets are indicated. Cells with significant enlargement of their MDC-positive compartment are indicated with arrows (b, c) Density of LC3 punctate staining, as a proxy for autophagy flux activation, was assessed from immunofluorescence microscopy images on HaCaT cells treated as indicated (see Materials and Methods). Exemplary cells with increased punctate pattern staining are highlighted with asterisks in panels at (b). Data plotted in (c) were derived from 50 cells from 3 independent biological replicates. ^∗∗∗^*p* ≤ 0.005.

We assessed the formation of autophagolysosomal vacuoles to infer canonical autophagy activation, indicative of cell adaption to generic stress. We first visualised the formation of autophagolysosomal vacuoles by MDC staining, as well as expansion of the lysosomal compartment as decorated by LysoTracker Green™. Consistent with a robust induction of xenotoxic stress, visual inspection revealed that exposure to PM_2.5_ increased the formation of autophagolysosomes (MDC+-positive mask) and was associated with an enlargement of the lysosomal compartment (LysoTracker Green+ mask) (Figure 3(a) second column of panels from left), both established features of prosurvival cell adaptive responses. Addition of Fernblock® further enhanced this expansion of the phagolysosomal compartment, and Fernblock® addition alone was also associated with a mild increase in the MDC/LysoTracker+ area (Figure 3(a), right panel columns). These observations are in agreement with experiments assessing relative density of LC3 puncta (indicative of *de novo* phagosome formation and maturation and activation of autophagy flux): Fernblock® increases basal autophagy flux in healthy cells and further enhances the activation provoked by PM_2.5_ exposure, consistent with its protective effect (Figures 3(b) and 3(c)).

These observations suggest that Fernblock® promotes the activation of survival pathways naturally employed by cells in the face of homeostasis challenges, and this induction correlates with its prosurvival and protective effects.

### 3.4. Fernblock® Upregulates NRF2 Basal Activity and Increases NRF2 Induction upon Exposure to PM_2.5_

**Figure 4:**
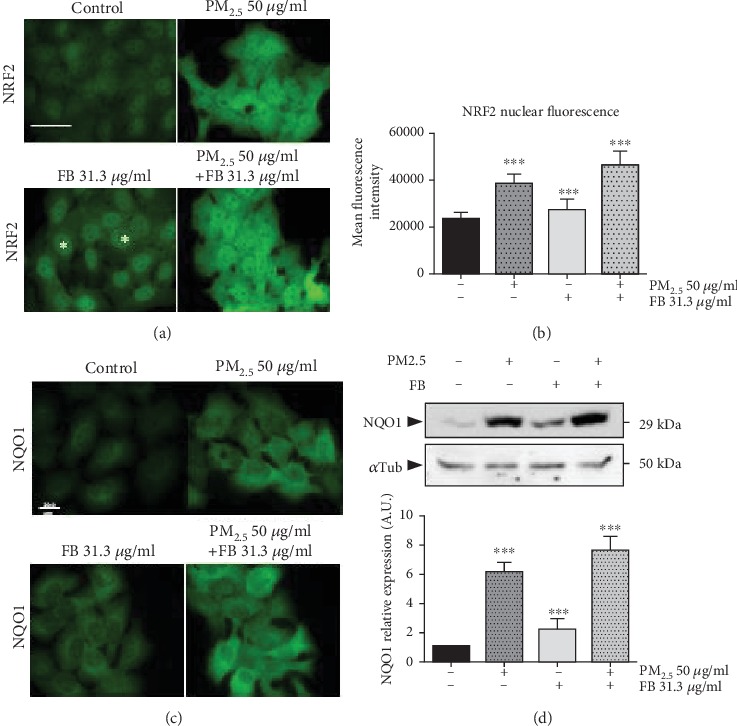
Fernblock® (FB) induces NRF2 activation and downstream transcription in keratinocytes. (a, b) NRF2 induction and activation was monitored in HaCaT cells by immunofluorescence microscopy across indicated treatment conditions. Scale bar (20 microns) is indicated. Exemplary cells with increased NRF2 staining specifically at the nuclear compartment are highlighted with asterisks in panel (a). Data plotted in (b) was derived from 50 cells from 3 independent biological replicates across conditions and expressed as normalized to untreated cells. (c, d) NQO1 induction downstream NRF2 was assessed in HaCaT cells by immunofluorescence microscopy (c) and immunoblotting (d, upper panel) across indicated conditions. (d, lower panel) Densitometric analysis of western blot data from 3 independent biological replicates is plotted as normalized to the signal observed in untreated cells. ^∗∗∗^*p* ≤ 0.005.

Because NRF2 activity is reported to be sensitive to xenotoxic stress from air pollutants [12, 13, 23], and our preliminary data demonstrated that the NRF2-dependent transcriptional pathway is triggered by treatment with Fernblock® (Figure 1), we moved to determine the relationship between Fernblock®-induced protection against PM_2.5_ toxicity and NRF2 regulation. Immunostaining revealed that unchallenged HaCaT keratinocytes in culture have low total and nuclear levels of NRF2 protein (Figures 4(a) and 4(b)). In accordance with the activation of an adaptive cytoprotective response, PM_2.5_ exposure alone leads to a robust increase in total NRF2 levels. Of note, Fernblock® exposure led *per se* to a mild increase specifically of NRF2 nuclear pools and further boosted NRF2 levels in cells exposed to PM_2.5_ (Figures 4(a) and 4(b)). Of note, Fernblock® did not entail the acute increase in cytoplasmic pools of NRF2 observed in cells exposed to PM_2.5_ (fig. [Supplementary-material supplementary-material-1], see Figure 4(a)).

**Figure 5:**
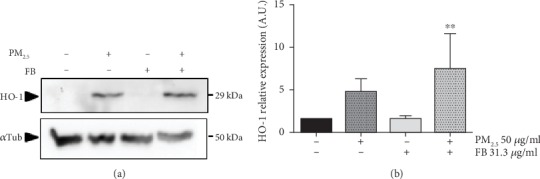
Fernblock® (FB) induces NRF2 activation and downstream transcription in keratinocytes. (a, b) HO-1 induction downstream NRF2 was assessed in HaCaT cells by immunoblotting (a, upper panel) across indicated conditions. (b) Densitometric analysis of western blot data from 3 independent biological replicates is plotted as normalized to the signal observed in untreated cells. ^∗∗^*p* ≤ 0.01.

We further assessed activation of the pathway downstream NRF2 by assessing the levels of two bona fide transcriptional targets of NRF2: NQO1 and HO-1. In line with our previous observations for NRF2 protein levels, PM_2.5_ exposure markedly upregulated NQO1 protein levels as assessed by immunofluorescent staining and western blot in HaCaT cells (Figures 4(c) and 4(d)). Fernblock® promoted a moderate increase in NQO1 levels in the absence of PM_2.5_ and significantly boosted their upregulation associated with PM_2.5_ exposure, in accordance with the measurements recorded for NRF2 protein (Figures 4(c) and 4(d)). HO-1 protein levels exhibited an analogous response and Fernblock® significantly enhanced the upregulation associated with PM_2.5_ exposure, as compared to the protective response observed in cells exposed to this particle pollution alone (Figures 5(a) and 5(b)).

## 4. Discussion

Air pollution is a major challenge to public health and well-being in modern societies, negatively impacting conditions such as cardiovascular disease, respiratory ailments and infections, and cancer. Thus, identifying compounds with protective properties against tissue damage due to this environmental source of xenotoxins is a priority for public health. An organ particularly exposed to damage from pollutants is the skin, and several cutaneous disorders are significantly influenced by environmental xenotoxins. Cellular mechanisms contrasting environmental pollutants include the AhR pathway, which positively regulates cyp450 detoxification systems and artemin [24]; the metal regulatory transcription factor-1 (MTF-1), which promotes the expression of antioxidant genes and metallothioneins in response to accumulation of heavy metals such as silver, cadmium, copper, or zinc [25]; and the conserved NRF2 pathway, which determines broad antioxidant and detoxifying transcriptional processes in response to different forms of stress leading to accumulation of ROS and electrophilic stress [9–13]. As such, NRF2 activity has emerged as a particularly attractive target for therapeutic and antiaging skin treatment [11, 26].

Natural compounds able to modulate these protective endogenous pathways are a promising option to reduce the impact of environmental pollution on skin. Several such compounds have in fact been identified which promote the NFR2 cytoprotective activities, including sulforaphane (from cruciferous vegetables) [21], curcumin (from *Curcuma longa*) [27], cinnamaldehyde (from cinnamon) [28], or tanshinones (from *Salvia miltiorrhiza*) [29]. Here, we provide evidence that Fernblock®, a standardized aqueous extract from *Polypodium leucotomos* leaves with proven photoprotective properties *in vivo* and *in vitro* against radiation damage in skin [16–19, 30], also induces protective mechanisms in an experimental model of fine particle PM_2.5_ air pollutants. Moreover, our studies provide novel evidence that the NRF2 transcriptional network is a relevant target upregulated by treatment with Fernblock® and demonstrate a close correlation between the upregulation of these antioxidant signalling pathways and the protective effect against damage from both UVB irradiation and fine particle pollutants. Due to their xenotoxic effect, PM_2.5_ exposure markedly increased total NRF2 protein levels as a defense mechanism, suggesting acute robust activation to counteract oxidative damage (Figures 4(a) and 4(b)). Fernblock® supplementation preferentially increased nuclear pools of NRF2. These observations support a hypothesis whereby Fernblock® is able to increase basal NRF2 activity levels in the absence of xenotoxic agent and further enhance its activation induced against PM_2.5_-induced oxidative stress, thus boosting the cell protection from damage. Several canonical routes downstream NRF2 (including autophagy and the direct NRF2 targets HO-1 and NQO1 which were demonstrated to be significantly impacted by Fernblock®) might play a role in the observed protection against PM_2.5_ damage. Of note, our observations of net protein level upregulation across conditions for the NRF2 targets HO-1 and NQO1 (Figures 4 and 5), together with our data showing a lack of impact on cell proliferation/viability (Figure 1), support a scenario whereby Fernblock® does not provoke cell toxicity and potentially positively regulates NRF2 through other mechanisms (*see below*). The crosstalk between NRF2 with other pathways such as AhR or NF-*κ*B-dependent inflammation networks involved in skin cell adaption to xenotoxins should also be taken into consideration [31, 32]. In addition to the cytoprotective routes driven by NRF2 in most cell types, an important factor to consider is the substantial impact NRF2 has on keratinocyte homeostasis through direct transcriptional control of different specialized structures, such as late cornified envelope 1 (LCE1) family members, keratins, and desmosomal components [33, 34].

Priming of autophagy, an important prosurvival and repairing mechanism [35], has also been demonstrated to be induced by treatment with Fernblock®. These studies thus also raise the important question as to how Fernblock® specifically interacts with cell metabolism, particularly interesting for its systemic (nutraceutical) applications. Modulation of key nodes of energy metabolism regulatory networks that also intersect with autophagy and ROS management, such as AMPK, is amenable though the use of well-established compounds such as metformin [36].

An important consideration that arises from these observations is the potential these mechanisms may have to slow skin aging. Loss of proteostasis and dysregulation of ROS levels and inflammation, decreased autophagy flux, and reduced NRF2 activity are all hallmarks of aging [37]. As stated above, NRF2 has also emerged as a relevant direct transcriptional regulator ensuring the expression of specialized keratinocyte components that decline with age, such as desmosomal proteins. Treatment with Fernblock® is effective for reducing radiation-induced cell senescence and age-associated damage in keratinocytes [17]. The present body of work further highlights the value of Fernblock® as an antiaging agent, whose beneficial effect might be the result of improved NRF2 signalling and autophagy. The potential of these mechanisms to attenuate accumulated oxidative stress and tissue damage is further exemplified by the reduction of UVB-induced melanogenesis, which is closely related to inflammation and oxidative stress in an evolutionarily conserved manner [38]. Adaptive pathways downstream NRF2, including Pi3/Akt signalling [39], autophagy itself [40, 41], and antioxidant effectors (potentially even by inhibiting the oxidative process of melanin biogenesis [42]), have been shown to contrast melanogenesis. Our observations support a model whereby Fernblock® reduces melanogenesis at least in part through upregulation of NRF2-dependent responses.

How does Fernblock® stimulate these prosurvival pathways? While other compounds currently being explored as “boosters” of NRF2 signalling, such as sulforaphane, exert their positive regulation of cytoprotection through canonical mechanisms [22], exact information regarding the molecular mechanisms by which Fernblock® modulates NRF2 and autophagy is lacking. A possible candidate mechanism could be the upregulation of sestrin family proteins, which have recently emerged as pivotal regulators upstream autophagy and ROS management in the cell [43]. A nonexclusive mechanism might be the modulation of cell metabolism and ROS production itself, currently not completely characterized for Fernblock®. It should be noted that autophagy is a relevant target of NRF2 itself: NRF2 drives the expression of essential autophagy regulators such as p62 and LAMP2A, and NRF2 deficiency reduces autophagy flux and favours the accumulation of proteotoxicity precursors [44, 45]. Our observations would agree with a speculative model whereby Fernblock® increases basal autophagy downstream enhanced NRF2 activity. Conversely, autophagy might also contribute to NRF2 activation through KEAP1 turnover, a mechanism likely underpinning the control of ROS through autophagy [46]. An intriguing additional potential mechanism both for Fernblock® antixenotoxic protection in general and for NRF2 regulation in particular may be embodied by the proteostatic network of the Heat Shock Protein response, which suppresses NF-*κ*B activity [47]. Because the identification of tools for promoting exogenous intervention of NRF2 signalling is an important objective across a broad range of fields in biomedicine, our findings highlight a potential use for Fernblock® as a natural compound able to upregulate this pathway, deserving of future research.

While the exact molecular mechanisms responsible for the loss of cell homeostasis upon exposure to PM_2.5_, and their modulation upon treatment with Fernblock®, remain to be fully characterized, our observations support a general model whereby Fernblock® reduces xenotoxicity by priming prosurvival antistress pathways, such as the NRF2 pathway and autophagy, thus reducing the oxidative stress and cell damage provoked by harmful agents such as PM_2.5_. Full characterization of the mechanisms modulated by Fernblock® may provide a framework for establishing personalized intervention and synergistic combinations that simultaneously boost these mechanisms as required.

## 5. Conclusions

**Figure 6:**
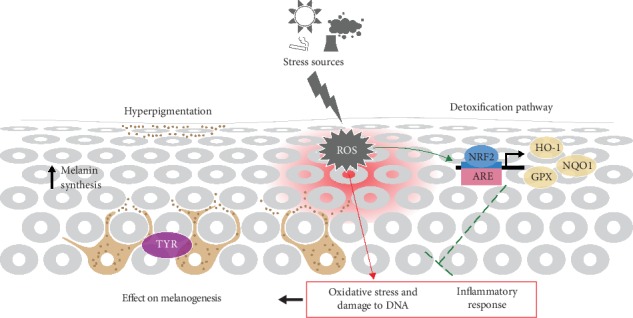
Scheme summarizing mechanisms identified in this study as potential sources of protection induced by Fernblock® against environmental damage of the skin.

Our results support NRF2 activation as a potential part of Fernblock®'s protective activity. Moreover, these studies have provided novel evidence that this protective activity is effective against not only oxidative stress derived from UVB radiation but also xenotoxic stress associated with exposure to fine particulate pollutants. Our observations strengthen the notion that Fernblock® can represent a functionally relevant and versatile compound effective against a wide range of environmental sources of tissue damage and aging and support its potential as a valuable tool (fully approved for human use) to elicit NRF2-dependent antistress responses across a wide range of conditions (graphical abstract, Figure 6).

## Data Availability

All cell biology, biochemistry and microscopy data supporting the findings of this report are included in this article. Source raw data for all quantitations is fully available upon request.

## Supplementary Materials

Supplementary Materials Figure S1: additional regional intensity analysis for NRF2 staining images from Figure 4(a) are shown. Data plotted are derived from the same image collections as in Figure 4(b). ^∗∗∗^*p* ≤ 0.005.
