# Supplementary figures and images for: Fernblock® Upregulates NRF2 Antioxidant Pathway and Protects Keratinocytes from PM2.5-Induced Xenotoxic Stress
Source: Oxid Med Cell Longev. 2020 Apr 14;2020:2908108. doi: 10.1155/2020/2908108 (PMC7181013; doi:10.1155/2020/2908108)

Figure S1

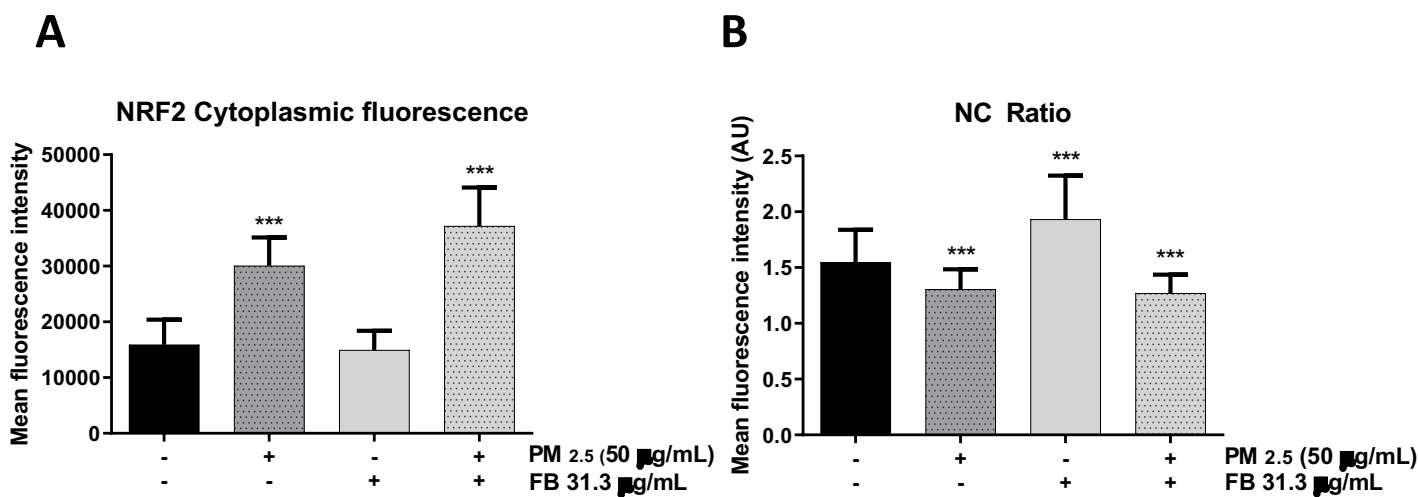

Supplement: Supplementary Materials — Figure S1: additional regional intensity analysis for NRF2 staining images from Figure 4(a) are shown. Data plotted are derived from the same image collections as in Figure 4(b). ∗∗∗p ≤ 0.005. [file 2908108.f1.pdf]
